# Habitual salt addition and self-reported cardiometabolic conditions among Saudi university students: a multi-region cross-sectional study

**DOI:** 10.3389/fnut.2026.1942220

**Published:** 2026-09-22

**Authors:** Abdulrahman I. Alaqil

**Affiliations:** Department of Physical Education, College of Education, King Faisal University, Al-Ahsa, Saudi Arabia

**Keywords:** added salt, body mass index, cardiometabolic disease, non-communicable disease prevention, NOVA classification, Saudi Arabia, ultra-processed foods, university students

## Abstract

**Background:**

Modifiable cardiometabolic risk factors include reported added salt, added sugar, and ultra-processed food (UPF) consumption. The combined dietary burden of UPF consumption and its associations with body mass index (BMI) and cardiometabolic disease remain unexamined among Saudi Arabian university students.

**Methods:**

This cross-sectional study included university students (*N* = 564) from four Saudi regions (March-May 2023). A validated semi-quantitative food frequency questionnaire assessed dietary exposure; a composite UPF score was derived from six NOVA-classified items. Multivariable linear regression examined diet-BMI associations; Firth-penalized logistic regression was pre-specified for the rare-event composite outcome (14 events; 2.5%) but did not converge to stable estimates; the reported disease association is therefore an unadjusted Fisher’s exact test. The multivariable BMI regression was adjusted for age, sex, academic level, college type, region, marital status, physical activity level (low/moderate/high), and total sedentary screen-time index.

**Results:**

Mean BMI was 22.22 ± 4.04 kg/m^2^; 64.0% of the sample were of normal weight. Habitual salt addition was reported by 87.1% of participants; high reported added salt (≥1 teaspoon/day) by 19.0%; high added sugar (≥3 teaspoon/day) by 16.0%. No dietary exposure was associated with BMI after adjustment (all *p* > 0.05). High reported added salt was significantly associated with prevalent self-reported cardiometabolic conditions (odds ratio 3.29; 95% confidence interval 1.04–10.42; *p* = 0.043); added sugar and UPF tertile were not. A sodium-smoking interaction on BMI was non-significant in the fully adjusted model (likelihood ratio test *p* = 0.053) and was treated as exploratory.

**Conclusion:**

This study provides the first multi-region evidence that high reported added salt was significantly associated with prevalent self-reported cardiometabolic conditions among Saudi university students despite a predominantly normal-weight profile. Given the small event count (*n* = 14) and cross-sectional design, these findings are exploratory only and do not support causal conclusions. Sodium reduction and salt literacy may warrant attention as NCD prevention strategies in this setting. Prospective studies with objective biomarkers are needed to establish causality.

## 1 Introduction

Non-communicable diseases (NCDs) are responsible for approximately 74% of all global mortality annually and remain the most common cause of preventable death worldwide (1, 2). Among the modifiable dietary risk factors implicated in the development of NCDs, three exposures are notable due to their mechanistic clarity and epidemiological consistency: excessive sodium intake, high added sugar consumption, and frequent consumption of ultra-processed food (UPFs) (3–6). Prospective cohort data and meta-analysis confirm independent dose-response associations between these exposures and the onset of obesity, hypertension, type 2 diabetes mellitus (T2DM), dyslipidemia, and non-alcoholic fatty liver disease (NAFLD), the same conditions that collectively constitute the cardiometabolic disease cluster (5, 7–10). Despite this convergent evidence, most extant studies examine individual dietary exposures in isolation; few studies have simultaneously assessed the joint contribution of these exposures in relation to a comprehensive chronic disease panel (11).

Saudi Arabia represents a high-burden setting in which these dietary exposures intersect with a rapidly evolving nutritional transition. Adult obesity prevalence increased from approximately 28.7% in 2015 to over one-third of the adult population by 2023–2024, accompanied by a substantial rise in T2DM, hypertension, and dyslipidemia (12–14). NCDs are now responsible for 73% of all premature deaths in Saudi Arabia; across Gulf Cooperation Council (GCC) countries, the NCD burden is estimated to cost 23.9 billion USD annually in direct healthcare expenditure and productivity loss (15, 16). Chiefly, these trends are attributed to traditional fiber-rich diets being displaced by energy-dense, sodium- and sugar-laden diets containing UPFs, a trend which has accelerated significantly since the 1990s (17). Recent Saudi-specific data confirm this picture: a NOVA-classified study of Saudi university students found that higher UPF intake was associated with a 30.2% higher BMI and more than twice the odds of abdominal obesity (17), while salt-related knowledge, attitude, and practice studies document that this population has persistently poor dietary practices and elevated habitual sodium intake (18).

University students represent a strategically important population for investigation in relation to NCDs. Transitioning to higher education constitutes a pivotal developmental window for young adults as, for the first time, their food choices become autonomous. This period is often characterized by deteriorations in dietary quality, and the habits formed during this period frequently remain in later adulthood (19, 20). Many studies highlight that students starting higher education consume more sugar-sweetened beverages (SSBs), eat less fruit and vegetables, and consume more fast food (21). In Saudi Arabia, these trends are compounded by high baseline NCD vulnerability (22). A recent cross-sectional study at the University of Hail revealed that 26.4% of undergraduates aged 21–26 were overweight and 17.1% were obese, a combined prevalence of 43.5% (23). Recent evidence suggests that young adults in Saudi Arabia have a poor awareness of cardiometabolic disease risk. A university-based survey reported that 56.2% of students lacked knowledge of NCDs and their associated risk factors (18). Despite this, extant Saudi student studies on this topic are limited to investigating individual dietary exposures or single health outcomes: none have simultaneously modeled the combined burden of sodium, sugar, and UPF intake against a multi-disease cardiometabolic panel.

Compounding these dietary risks, tobacco use, particularly shisha (waterpipe) smoking, remains highly prevalent among Eastern Mediterranean young adults and has emerged as a common form of tobacco consumption among Saudi Arabian university students (24). A single shisha session (approximately 40 min) delivers the same amount of nicotine as smoking 100 or more cigarettes, meaning that users are exposed to elevated levels of carbon monoxide and toxic aldehydes that exert pro-inflammatory and atherogenic effects (25). In GCC cohorts, shisha smoking is linked to a non-significant trend toward increased cardiovascular risk (26) and may also synergistically amplify the cardiometabolic harm of unhealthy dietary habits through shared oxidative and inflammatory pathways. Sociodemographic factors further shape both dietary exposure and disease risk: globally, lower household income is consistently associated with greater reliance on energy-dense UPFs, partly due to cost constraints and accessibility (27, 28). In Saudi Arabia, sex simultaneously shapes dietary habits, nutritional adequacy, and tobacco exposure (29). To date, however, no Saudi Arabian studies have formally examined whether smoking status moderates the diet-disease relationship, nor attempted to stratify these associations by both sex and income within a single, multi-region student sample. These gaps limit the evidence base for designing and implementing equitable nutrition interventions to target the sociodemographic factors associated with the early onset of NCDs among Saudi university students.

The extant literature on NCD risk among Saudi university students remains patchy. To date, no studies have collectively examined reported added salt, added sugar consumption, and UPF consumption in relation to a multi-disease cardiometabolic panel that includes T2DM, hypertension, dyslipidemia, cardiovascular disease (CVD), and NAFLD. To address this gap, the current study adopted a multi-region, cross-sectional design to (i) assess the prevalence and sociodemographic predictors of these three dietary exposures, and (ii) determine their independent and combined associations with BMI and cardiometabolic disease risk, stratified by sex and household income, along with smoking status as a potential moderating factor.

## 2 Materials and methods

### 2.1 Study design and setting

This cross-sectional study was conducted between March and May 2023 and reported in accordance with the Strengthening the Reporting of Observational Studies in Epidemiology (STROBE) guidelines (30). Coordinated by the Deanship of Scientific Research, data was collected via a self-administered online questionnaire distributed via Google Forms to participants’ institutional email accounts. Participation was voluntary and anonymous; completion of the questionnaire was deemed to imply informed consent. Ethical approval was granted by the Institutional Review Board of King Faisal University, Saudi Arabia (KFU-REC-2022-OCT-ETHICS202); this study was conducted in accordance with the Declaration of Helsinki.

### 2.2 Participants and sampling

The target population comprised currently enrolled students at Saudi Arabian universities aged > 18 across the following four regions: Makkah (39.0%), the Eastern Province (29.4%), Riyadh (28.5%), and other regions, including e.g., Asir, Al-Madinah, and Hail (3.0%). Systematic random sampling was used to select three colleges per university. Students of health and medical sciences, arts and humanities, and science and engineering were recruited; 52.0% of participants were studying health-related subjects while 47.7% were studying non-health sciences. Within each college, one male and one female class were sampled from each of the four undergraduate years and the two postgraduate years, with all students in the selected classes invited to participate. The minimum required sample size was determined to be 384, calculated using 95% confidence, 5% margin of error, and 50% expected prevalence of T2DM risk factors. The final sample (*N* = 564; males: *n* = 136; 24.1%; females: 428; 75.9%) exceeded the sample requirement, and a 100% completion rate was confirmed through classroom-based administration. The female predominance (75.9%) reflects enrolment patterns in the sampled health sciences colleges rather than a sampling bias (31). No formal sampling frame or invitation list was maintained; accordingly, a conventional non-response rate cannot be calculated. The denominator for the completion rate is the number of students present in selected classes on the day of data collection.

### 2.3 Survey instrument

A structured Arabic-language questionnaire was used to collect data across four domains: (i) sociodemographic characteristics and anthropometrics; (ii) tobacco use; (iii) physical activity and sedentary behaviors; and (iv) dietary intake. The dietary component was based on an adapted semi-quantitative food frequency questionnaire (SFFQ) validated for use in Saudi Arabian populations (32). The SFFQ comprised 66 food and beverage variables (53 primary items and 13 sub-items), each scored on an eight-level frequency scale from never to more than three times per day. Beverage intake was assessed using a daily cups-or-glasses scale; an open-ended item captured the consumption of foods not listed in the structured questionnaire.

### 2.4 Variable definitions

#### 2.4.1 Sociodemographic and anthropometric variables

Sociodemographic variables included sex, age group, nationality, marital status, number of children, monthly household income, academic level (undergraduate year 1–4, master’s, Ph.D.), and college type (health sciences or non-health sciences). Income was categorized into one of six bands (from ≤3,000 to ≥23,000 SAR/month) before being dichotomized (low income: ≤8,000 SAR; middle-to-high income: >8,000 SAR) to enable stratified analyses and consistent with income stratification used in Saudi health research (28, 33). Body weight and standing height data were self-reported; BMI was calculated as weight (kg) divided by height squared (m^2^) and classified as per the following WHO criteria: underweight (<18.5), normal weight (18.5–24.9), overweight (25.0–29.9), and obese (≥30.0 kg/m^2^) (34). Six participants who provided incomplete anthropometric data were excluded from the BMI analyses.

#### 2.4.2 Dietary exposures

Three primary dietary exposures were assessed: reported added salt, added sugar consumption, and UPF consumption. Reported added salt was measured using a binary question asking whether participants habitually added salt to food (yes/no) and an ordinal item estimating the quantity of salt added daily (six levels: <1/4 to >2 teaspoons/day). Of the 491 participants who added salt to their food (87.1%), 119 (24.2%) were unable to quantify their salt intake; these were treated as missing data and addressed using multiple imputations. To enable regression analyses, reported added salt was dichotomized as high (≥1 teaspoon/day) or low. This threshold corresponds to approximately 2,300 mg of sodium (5.8 g of salt per day), meaningfully exceeding the WHO recommended maximum of 5 g/day (35), and represents the clinically relevant cut-point for excess discretionary sodium intake. Added sugar intake was measured as the number of teaspoons of sugar added to hot beverages or breakfast cereal per day (0 to ≥5 teaspoons) and dichotomized as high (≥3 teaspoons/day) or low, corresponding to the upper tertile of the sample distribution. This variable represents sugar added to hot beverages or breakfast cereal only, not total free sugar intake from all sources. As total energy intake was not assessed by the SFFQ used, this threshold could not be related to the WHO recommendation of <10% of total daily energy from free sugars; it was therefore selected as a pragmatic, distribution-based cut-point rather than a clinically established limit.

Ultra-processed food consumption was operationalized using six food items classified according to the NOVA framework (36): potato chips and crisps; sugar-sweetened and energy beverages; ready-made processed foods (including frozen pizza, processed chicken fingers, and ready-to-eat fried burgers); sausages and cold cuts; broth cubes and soup sachets; and pickled or dried vegetables. Each of these items was classified as NOVA Group 4 based on their typical commercial formulation in Saudi Arabia, which involves industrial additives, flavor enhancers, and preservatives, consistent with prior Saudi NOVA-based dietary research (17). Each item was scored on an eight-level frequency scale from never (0) to more than three times per day (7). Broth cubes were assessed as weekly quantity (0–6 cubes) and linearly rescaled to 0–7; 57 participants (10.1%) who selected “not sure” were replaced with the item mean. A composite UPF score was calculated by summing all six items (range 0–35; mean ± SD: 9.20 ± 6.66) and categorizing them into the following tertiles: low (≤5.2), moderate (5.2–11.0), and high (>11.0), as no participant scored the maximum on all items simultaneously. The six-item proxy captures common high-prevalence UPF categories in Saudi dietary patterns but does not represent the full NOVA Group 4 spectrum, which encompasses dozens of product categories.

#### 2.4.3 Cardiometabolic outcome

Chronic disease history was assessed using a self-reported checklist of nine conditions (T2DM, hypertension, kidney failure, CVD, stroke, hypercholesterolemia, hypertriglyceridemia, NAFLD, and others). Given the low individual event rates in this young sample (e.g., T2DM: 1.1%; hypertension: 0.2%; CVD: 0.4%), individual conditions were reported descriptively only. A binary composite cardiometabolic outcome, namely the presence of at least one of the following conditions: T2DM, hypertension, CVD, hypercholesterolemia, hypertriglyceridemia, or NAFLD (*n* = 14; 2.5%), was used as the primary binary outcome in regression analyses, this being a pragmatic analytical choice driven by event sparsity rather than clinical homogeneity, with BMI serving as the continuous primary outcome.

#### 2.4.4 Tobacco use

Current smoking status was recorded as a binary variable (yes/no). Smokers identified their tobacco product(s) from five options: mu’assel, cigars, combustible cigarettes, shisha or hookah, and electronic cigarettes. To enable moderation analyses, smoking was treated as binary (smoker or non-smoker); a four-category variable (i) non-smoker; (ii) combustible cigarette user; (iii) electronic cigarette user; (iv) waterpipe user (shisha or mu’assel) was used in the secondary analyses.

#### 2.4.5 Covariate

Physical activity was assessed using six items adapted from the IPAQ-SF (37, 38) to capture the frequency (days per week) and duration (minutes per day) of vigorous activity, moderate activity, and walking that participants had engaged in during the preceding 7 days. Duration was recorded using a five-level ordinal scale; midpoint values (20, 45, 90, 150, and 210 min) were assigned to each category to enable computation of total weekly metabolic equivalent of task (MET) minutes as per the IPAQ-SF scoring guidelines (vigorous: 8.0 METs; moderate: 4.0 METs; walking: 3.3 METs) (38). Physical activity level was then categorized as low (<600 MET-min/week), moderate (600–2,999 MET-min/week), or high (≥3,000 MET-min/week). Sedentary behavior was quantified across six daily screen-time domains (academic use, television, mobile phone, laptop, gaming, and other leisure), with each scored from 0 to 5 (none to >4 h/day) to produce a total sedentary screen-time index (range 0–30; mean ± SD: 12.7 ± 4.9) (39). Both physical activity category and sedentary screen-time index were then included as covariates in the multivariable regression models.

#### 2.4.6 Statistical analysis

All analyses were performed using IBM’s SPSS Statistics Package (version 29.0; IBM Corp., Armonk, NY, USA). Continuous variables are reported as mean ± standard deviation (SD) or median (interquartile range); categorical variables as frequency and percentage. Between-group differences were assessed as appropriate using independent *t*-tests or Mann-Whitney U tests for continuous variables or chi-square or Fisher exact tests for categorical variables. Associations between dietary exposures and BMI were examined using multivariable linear regression, reported as unstandardized β coefficients with 95% confidence intervals (CIs), adjusted for age, sex, academic level, college type, region, marital status, and physical activity (low/moderate/high as per the IPAQ-SF guidelines), and total sedentary screen-time index. Due to the low event rate of the binary composite outcome (*n* = 14; 2.5%), Firth-penalized logistic regression was the pre-specified primary method (40, 41); however, owing to sparse cell counts across the multiple covariates, the model did not yield stable adjusted estimates. Accordingly, the association between reported added salt and the composite cardiometabolic outcome is reported as an unadjusted Fisher’s exact test; the added sugar association is also reported using Fisher’s exact test; and the UPF tertile association is reported using chi-square. No covariates were included in Table 1 analyses. These analyses are unadjusted and should be interpreted as exploratory only. The moderating effect of smoking status on the diet-BMI association was assessed via a product interaction term (dietary risk index × smoking status), tested using the likelihood ratio test and reported as exploratory given the small smoker subgroup. Stratified analyses by sex and household income were conducted *a priori*. A two-tailed *p* < 0.05 was considered statistically significant. Missing data for added salt quantity (119/491 salt users; 21.1% of salt users; 24.2% of those who added salt) were handled using multiple imputation by chained equations (MICE; 20 datasets, pooled using Rubin rules) (42). The imputation model included all analytical variables plus auxiliary predictors (smoking status, sex). After imputation of salt quantity and listwise deletion for remaining missing covariates, the primary regression model included *n* = 557 participants. Missing physical activity duration values (6.2%–16.5%) arising from skip logic were set to zero, consistent with the questionnaire skip pattern indicating no activity on those days; missing broth cube values (10.1%, recorded as “not sure”) were replaced with the item mean as a conservative central estimate. All remaining variables had <2% missing data and were handled using complete-case analysis. A complete-case sensitivity analysis (participants with all variables observed including salt quantity; *n* = 440) confirmed materially consistent results (see Table 2).

**TABLE 1:** Unadjusted exploratory association between reported dietary exposures and any prevalent self-reported cardiometabolic condition (total: *N* = 564; 14 events; added salt analysis: *n* = 445 with quantifiable intake; 12 events among those with known salt status; 2 disease cases are in the 119 participants with unknown salt quantity).

Dietary exposure	Cases/*n*	Prevalence (%)	OR (95% CI)	*P*-value
Reported added salt				
Low added salt (ref)	6/338	1.8%	1.00 (ref)	–
High added salt (≥1 tsp/day)	6/107	5.6%	3.29 (1.04–10.42)	0.043†
Added sugar intake				
Low sugar (ref)	13/474	2.7%	1.00 (ref)	–
High sugar (≥3 tsp/day)	1/90	1.1%	0.45 (0.06–3.53)	0.700
UPF consumption tertile				
Low (≤5.2; ref)	7/157	4.5%	–	0.222‡
Moderate (5.2–11.0)	2/145	1.4%	–	
High (>11.0)	3/143	2.1%	–	

All analyses are unadjusted and exploratory. OR, odds ratio from Fisher’s exact test (added salt, sugar) or chi-square (UPF); 95% CIs from log-odds method.

†*p* < 0.05; based on 6 events in the high-salt group; salt analysis uses *n* = 445 with quantifiable intake, of whom 12 have disease; 2 disease cases are among the 119 with unknown salt quantity and excluded from the salt analysis.

‡Chi-square; UPF and sugar analyses use full sample (*N* = 564; 14 events). Firth penalized logistic regression was pre-specified but did not yield stable estimates with 14 events; results are therefore unadjusted. All conditions are self-reported and not clinically verified. Reverse causation is plausible.

**TABLE 2:** Multivariable linear regression: association between dietary exposures and BMI (primary model: *n* = 557 after multiple imputation; complete-case sensitivity: *n* = 440).

Variable	β	95% CI	*P*-value
Primary dietary exposures			
High reported added salt (ref: low)	0.14	−0.77 to 1.05	0.761
High added sugar intake (ref: low)	−0.32	−1.26 to 0.61	0.498
UPF moderate tertile (ref: low)	−0.27	−1.08 to 0.54	0.515
UPF high tertile (ref: low)	−0.36	−1.20 to 0.48	0.403
Covariates			
Sex: female (ref: male)	−0.61	−1.53 to 0.31	0.194
PA level: moderate (ref: low)	−0.53	−1.49 to 0.42	0.275
PA level: high (ref: low)	−0.05	−1.09 to 0.99	0.925
Sedentary index (per unit increase)	0.06	−0.01 to 0.13	0.118

Table reports the primary model after multiple imputation of added salt quantity (MICE, 20 datasets; *n* = 557; R^2^ = 0.114; adjusted R^2^ = 0.063). A complete-case sensitivity analysis restricted to participants with all variables observed, including salt quantity (*n* = 440), produced materially consistent estimates (added salt β = 0.14, *p* = 0.761; R^2^ = 0.144; adjusted R^2^ = 0.083). Models adjusted for age group, sex, academic level, college type, administrative region, marital status, and physical activity level. β, unstandardized regression coefficient; CI, confidence interval; UPF, ultra-processed food. Reference category for UPF is low tertile (score ≤ 5.2). Complete-case sensitivity analysis (*n* = 440): added salt β = 0.14, *p* = 0.761; R^2^ = 0.144, consistent with the primary imputed model.

## 3 Results

The total sample of Saudi Arabian university students (*N* = 564; female: 75.9%; mean BMI 22.22 ± 4.04 kg/m^2^) was included in the analysis. Most participants were single (93.4%), enrolled in health sciences colleges (52.0%), and primarily from Makkah (39.0%), the Eastern Province (29.4%), and Riyadh (28.5%). Overall, 64.0% of the samples were normal weight, 17.2% were underweight, 12.9% were overweight, and 4.8% were obese. Current tobacco use was reported by 7.6% of the sample, with electronic cigarettes (39.5% of smokers) and combustible cigarettes (37.2%) being most common. Full socio-demographic and clinical characteristics, stratified by sex, are presented in Table 3.

**TABLE 3:** Sociodemographic, anthropometric, and self-reported clinical characteristics of Saudi university students, stratified by sex (*N* = 564).

Characteristic	Total (*N* = 564) *n* (%)	Male (*n* = 136) *n* (%)	Female (*n* = 428) *n* (%)	*P*
Age group				
<20 years	164 (29.1%)	56 (41.2%)	108 (25.2%)	0.008*
20–25 years	385 (68.3%)	77 (56.6%)	308 (72.0%)	
26–30 years	7 (1.2%)	1 (0.7%)	6 (1.4%)	
>30 years	8 (1.4%)	2 (1.5%)	6 (1.4%)	
Marital status				
Single	527 (93.4%)	132 (97.1%)	395 (92.3%)	0.168
Married	26 (4.6%)	2 (1.5%)	24 (5.6%)	
Divorced/widowed/separated	11 (2.0%)	2 (1.5%)	9 (2.1%)	
Monthly household income (SAR)				
≤3,000	101 (17.9%)	40 (29.4%)	61 (14.3%)	
3,001–8,000	128 (22.7%)	26 (19.1%)	102 (23.8%)	
8,001–13,000	129 (22.9%)	27 (19.9%)	102 (23.8%)	
13,001–18,000	85 (15.1%)	21 (15.4%)	64 (15.0%)	
18,001–23,000	62 (11.0%)	15 (11.0%)	47 (11.0%)	
≥23,000	59 (10.5%)	7 (5.1%)	52 (12.1%)	
Low income (≤8,000 SAR)	229 (40.6%)	66 (48.5%)	163 (38.1%)	0.059
Academic level				
Year 1	97 (17.2%)	56 (41.2%)	41 (9.6%)	<0.001*
Year 2	188 (33.3%)	20 (14.7%)	168 (39.3%)	
Year 3	172 (30.5%)	46 (33.8%)	126 (29.4%)	
Year 4	96 (17.0%)	12 (8.8%)	84 (19.6%)	
Postgraduate	11 (2.0%)	2 (1.5%)	9 (2.1%)	
College type				
Health sciences	293 (52.0%)	94 (69.1%)	199 (46.5%)	<0.001*
Non-health sciences	269 (47.7%)	40 (29.4%)	229 (53.5%)	
Body mass index				
Mean ± SD (kg/m^2^)	22.22 ± 4.04	22.03 ± 3.56	22.28 ± 4.18	0.876
Underweight (<18.5)	97 (17.2%)	24 (17.6%)	73 (17.1%)	
Normal weight (18.5–24.9)	361 (64.0%)	85 (62.5%)	276 (64.5%)	
Overweight (25.0–29.9)	73 (12.9%)	20 (14.7%)	53 (12.4%)	
Obese (≥30.0)	27 (4.8%)	4 (2.9%)	23 (5.4%)	
Tobacco use				
Non-smoker	521 (92.4%)	113 (83.1%)	408 (95.3%)	<0.001*
Current smoker	43 (7.6%)	23 (16.9%)	20 (4.7%)	
Cigarettes	16 (37.2%)†	12 (52.2%)†	4 (20.0%)†	
E-cigarettes	17 (39.5%)†	8 (34.8%)†	9 (45.0%)†	
Mu’assel	8 (18.6%)†	6 (26.1%)†	2 (10.0%)†	
Shisha/hookah	3 (7.0%)†	2 (8.7%)†	1 (5.0%)†	
Self-reported cardiometabolic disease				
T2DM	6 (1.1%)	3 (2.2%)	3 (0.7%)	
Hypertension	1 (0.2%)	1 (0.7%)	0 (0.0%)	
Cardiovascular disease	2 (0.4%)	1 (0.7%)	1 (0.2%)	
Hypercholesterolemia	5 (0.9%)	0 (0.0%)	5 (1.2%)	
Any cardiometabolic condition	14 (2.5%)	5 (3.7%)	9 (2.1%)	0.312

*P*-values from chi-square test for categorical variables and Mann-Whitney U test for BMI.

*Statistically significant (*p* < 0.05).

†Percentages for tobacco product type are calculated among current smokers only (*n* = 43). BMI categories based on WHO criteria. T2DM, type 2 diabetes mellitus; SAR, Saudi Arabian Riyal; SD, standard deviation. All disease conditions are self-reported and not clinically verified.

Most participants (87.1%) reported adding salt to food, of whom 19.0% of the total sample consumed one or more teaspoons of salt per day. High added sugar intake (≥3 teaspoons/day) was reported by 16.0% of participants. Potato chips were the most frequently consumed UPF item (64.5% weekly), followed by sugar-sweetened and energy beverages (45.0%), and ready-made processed foods (41.3%). The UPF composite score ranged from 0 to 35 (mean ± SD: 9.20 ± 6.66). Health sciences students showed marginally higher high-sodium prevalence (25.1%) than non-health sciences students (23.1%, *p* = 0.623) and slightly higher mean UPF scores (9.59 vs. 8.81, *p* = 0.235), with neither difference reaching statistical significance; this comparison cannot establish whether health-sciences training confers a protective dietary effect. Table 4 presents dietary exposures stratified by sex and income; no statistically significant differences were evident across any dietary variable (all *p* > 0.05).

**TABLE 4:** Dietary exposure prevalence by sex and monthly household income among Saudi university students (*N* = 564).

Variable	Total (*N* = 564)	Male (*n* = 136)	Female (*n* = 428)	*P*	Low income (*n* = 229)	Mid–high income (*n* = 335)	*P*
Reported sodium intake							
Add salt to food, *n* (%)	491 (87.1%)	122 (89.7%)	369 (86.2%)	0.363	198 (86.5%)	293 (87.5%)	0.826
High added salt (≥1 tsp/day), *n* (%)	107 (19.0%)	18 (13.2%)^b^	89 (20.8%)^b^	0.069	38 (16.6%)	69 (20.6%)	0.304
Added sugar intake							
High sugar (≥3 tsp/day), *n* (%)	90 (16.0%)	25 (18.4%)	65 (15.2%)	0.452	38 (16.6%)	52 (15.5%)	0.823
Ultra-processed food (UPF) consumption							
UPF composite score, mean ± SD	9.20 ± 6.66	9.52 ± 6.98	9.09 ± 6.56	0.450	9.59 ± 7.18	8.93 ± 6.28	0.497
Low tertile (≤5.2), *n* (%)	195 (34.6%)	44 (32.4%)	151 (35.3%)	0.247^c^*	80 (34.9%)	115 (34.3%)	0.207^c^*
Moderate tertile (5.2–11.0), *n* (%)	194 (34.4%)	42 (30.9%)	152 (35.5%)		70 (30.6%)	124 (37.0%)	
High tertile (>11.0), *n* (%)	175 (31.0%)	50 (36.8%)	125 (29.2%)		79 (34.5%)	96 (28.7%)	

*P*-values for continuous variables from Mann-Whitney U test; for categorical variables from chi-square test.

^a^Percentage among the total sample (*N* = 564).

^b^Among salt users, high sodium prevalence was 17.0% in males and 26.3% in females.

^c^*P*-value for UPF tertile distribution (chi-square across three categories). tsp, teaspoon; UPF, ultra-processed food; SAR, Saudi Arabian Riyal.

In unadjusted analyses, mean BMI did not differ significantly between participants with high versus low reported added salt (22.43 ± 4.25 vs. 22.19 ± 3.89 kg/m^2^; *p* = 0.781), high versus low added sugar intake (22.01 ± 4.16 vs. 22.26 ± 4.02 kg/m^2^; *p* = 0.448), or across UPF tertiles (Kruskal–Wallis *p* = 0.515). The multivariable linear regression adjusted for age, sex, academic level, college type, region, marital status, physical activity level (IPAQ-SF categories), and sedentary screen-time index (primary model after multiple imputation: *n* = 557; complete-case sensitivity: *n* = 440; Table 2) revealed that none of the three dietary exposures was independently associated with BMI in either analysis. The sedentary screen-time index indicated a borderline positive association with BMI (β = 0.07; 95% CI: −0.00 to 0.15; *p* = 0.059). The model accounted for 14.6% of the variance in BMI (adjusted R^2^ = 0.086).

A total of 14 participants (2.5%) reported having at least one cardiometabolic condition: T2DM (*n* = 6; 1.1%), hypercholesterolemia (*n* = 5; 0.9%), CVD (*n* = 2; 0.4%), and hypertension (*n* = 1; 0.2%). As shown in Table 1, high reported added salt was significantly associated with any prevalent self-reported cardiometabolic condition compared with low or no added salt (OR = 3.29; 95% CI: 1.04–10.42; *p* = 0.043; unadjusted; exploratory). Neither high added sugar (OR = 0.45; 95% CI: 0.06–3.53; *p* = 0.700) nor UPF tertile (chi-square *p* = 0.222) was significantly associated with the composite outcome. Given the small number of events (*n* = 14), all associations should be regarded as preliminary as they require confirmation by studies with larger samples.

The interaction between high reported added salt and smoking status on BMI was not statistically significant in the fully adjusted model (β = −3.12; likelihood ratio test χ^2^ = 3.73; *p* = 0.053). This finding should be treated as strictly exploratory given the small smoker subgroup (*n* = 43) and should NOT be interpreted as a substantive finding. No smokers in this sample reported a cardiometabolic diagnosis; accordingly, the moderation of the disease composite outcome was not estimable. Sex- and income-stratified regression analyses yielded no statistically significant diet-BMI associations in any sub-group, although a non-significant trend toward lower BMI with higher UPF consumption was observed in the low-income stratum (β = −1.35; 95% CI: −2.89 to 0.18; *p* = 0.085). All subgroup findings should be treated as exploratory.

## 4 Discussion

The most clinically relevant finding is the threefold elevation in the odds of cardiometabolic disease among participants reporting high added salt compared with those reporting low or no added salt (unadjusted; Fisher’s exact test; *n* = 445; 14 events) (OR = 3.29; 95% CI: 1.04–10.42; *p* = 0.043). Given the small event count and cross-sectional design, this finding should not be interpreted as evidence of causation. It was observed in a sample in which 87.1% habitually added salt to food, and 19.0% consumed one or more teaspoons of salt per day, levels that exceed the WHO’s recommended maximum salt intake (5 g per day) (35). This finding is consistent with a substantial mechanistic and prospective literature linking excessive sodium intake to the risk of hypertension, endothelial dysfunction, and CVD (43). A large UK biobank study demonstrated a dose-response relationship between habitually adding salt to food and incident cardiovascular disease and all-cause mortality (44). The current study extends this finding to the young Saudi student population in whom cardiometabolic conditions were already detectable. As true added salt exposure is likely underestimated [nearly a quarter of salt users could not quantify their daily intake and habitual salt users systematically under-report relative to objective 24-h urinary measures (45, 46)], the direction of misclassification bias is unknown.

By contrast, none of the three dietary exposures investigated were independently associated with BMI after multivariable adjustment, a null pattern that was interpretable given the sample profile and study design. With 64.0% of participants classified as normal weight and a mean BMI of 22.22 kg/m^2^, the restricted variance in the primary outcome inherently limits statistical power to detect modest diet-adiposity associations, a limitation also documented in comparable normal-weight university cohorts (47, 48). Additionally, cross-sectional research designs are vulnerable to reverse causality, whereby already overweight individuals may have modified their diets prior to assessment, thereby attenuating any observable associations (49). The counterintuitive inverse trend between the high UPF tertile and BMI (β = −0.76; *p* = 0.107), consistent with findings in other student population samples with favorable average BMI (47), may reflect either dietary restraint among weight-conscious participants or the insensitivity of BMI as an adiposity outcome in this age group; however, these remain speculative explanations. UPF consumption adversely affects body fat percentage and visceral adiposity before increasing BMI (50), suggesting that body composition outcomes would be more informative endpoints in future work. The choice of UPF classification system further complicates interpretation: the direction and magnitude of UPF-cardiometabolic associations vary substantially across NOVA, IARC, and UNC frameworks (51), and the six-item proxy employed in the current study, while NOVA-aligned, does not capture the full Group 4 spectrum.

Sex- and income-stratified analysis did not highlight any significant diet-BMI associations. Health sciences students showed no significant dietary advantages over non-health sciences students (high-sodium prevalence: 25.1% vs. 23.1%, *p* = 0.623; UPF scores: 9.59 vs. 8.81, *p* = 0.235), though this cross-sectional comparison cannot establish whether health-sciences training has or lacks a protective effect. The absence of income-related dietary disparities, which runs counter to international evidence linking lower socioeconomic position to greater reliance on UPFs (28), may reflect the relatively homogeneous food environment among enrolled Saudi university students, where institutional food availability and peer norms may exert stronger influence than individual purchasing constraints; however, this explanation is speculative. The sodium × smoking interaction in relation to BMI, which attenuated to non-significance in the fully adjusted model (likelihood ratio test *p* = 0.053), whereby smokers with high reported added salt had lower BMI, was directionally consistent with the appetite-suppressant effects of nicotine observed in the cross-sectional analysis (52). However, no cardiometabolic cases were present among the 43 smokers in the sample; this interaction, however, remains strictly hypothesis-generating and requires further confirmation using larger, adequately powered samples.

These findings have direct implications for NCD prevention policy among Saudi university students and Saudi Arabian university campus health practices. The high prevalence of habitual salt addition, the association with NCDs at this early life stage, and the widespread inability of students to self-monitor their salt addition collectively points to sodium reduction initiatives as a priority dietary intervention area for this population. Such initiatives may be justified on the basis of the broader evidence base linking sodium to cardiometabolic disease, rather than on the present cross-sectional and exploratory finding alone. Salt literacy programs may incorporate practical tools such as providing graduated salt spoons, visual portion guides, and requiring sodium labeling on canteen menus, all of which are low-cost, high-reach strategies well-suited to university settings. Such initiatives are likely to be well supported by Saudi Vision 2030 and the National Transformation Program, which provide an favorable policy framework for enacting such preventive health initiatives (53). Although associations between UPF and SSB intake and the prevalence of NCDs were not statistically significant in this cross-sectional sample, the high frequency of consumption of these items [e.g., UPFs (chips): 64.5% weekly; SSBs: 45.0% weekly] represents a risk trajectory that warrants attention in campus food environment planning. Such reform could include implementing the Saudi Healthy Plate 2024 framework in university canteens (54) to mitigate the accumulating cardiometabolic risks over the following decades. The significantly higher tobacco use among males compared to females (16.9% vs. 4.7%), combined with the sodium-smoking interaction finding, supports the development of initiatives that integrate dietary counseling with tobacco cessation services in university health programs targeting male students. Males remain systematically under-represented in Saudi dietary research and require dedicated surveillance investment.

The notable methodological strengths of this study are its multi-region, multi-college design, which sampled both health and non-health science students across all academic levels, and the application of the Saudi-validated SFFQ (32); Firth-penalized logistic was pre-specified for the rare-event outcome but did not converge, and the disease association was therefore reported as an unadjusted, exploratory analysis using Fisher’s exact test. Nevertheless, the present study’s findings must be interpreted with caution considering the following limitations. First, the cross-sectional research design precludes causal inference regarding the associations between reported added salt, added sugar intake, and UPF consumption, and BMI and cardiometabolic disease. Second, the use of self-reported anthropometric data introduces systematic error, particularly weight underestimation among females and height overestimation in both sexes (55), and the low disease prevalence (2.5%; 14 events) substantially limits power for binary outcome analysis, as wide confidence intervals were identified even for the significant association of reported added salt. Third, the six-item UPF proxy does not represent the full NOVA Group 4 spectrum. The composite cardiometabolic outcome combines conditions that are clinically heterogeneous in pathophysiology (T2DM, hypertension, CVD, hypercholesterolemia, hypertriglyceridemia, NAFLD); this pragmatic grouping, necessitated by the small number of individual events, limits interpretation of the pooled association. Important confounding variables were not assessed, including total energy intake (not available from the SFFQ used), potassium intake, medication use, family history of cardiometabolic disease, sleep, and stress; the absence of total energy intake in particular limits interpretation of the sugar and UPF analyses. Fourth, the predominance of female participants (75.9%) and the geographic focus on only three regions of Saudi Arabia limit the generalizability of the results to other contexts. Fifth, reported added salt was assessed by self-report only, without objective biomarkers such as 24-h urinary sodium excretion, and habitual salt users are known to systematically underestimate their intake relative to urinary measures (46). Finally, the 100% response rate, achieved through classroom-based sampling, may have introduced selection bias: academically engaged students with atypical dietary profiles may have been more likely to participate. To overcome these shortcomings, future longitudinal studies featuring objective sodium biomarkers, validated body composition measures, and comprehensive NOVA dietary assessment are required to establish causal pathways in this population.

## 5 Conclusion

High reported added salt was significantly associated with prevalent self-reported cardiometabolic conditions in this exploratory, unadjusted analysis. These findings are hypothesis-generating only; the cross-sectional design, self-reported measures, and small event count (14 cases) preclude causal conclusions. Added sugar and UPF consumption were not independently associated with BMI or NCDs in the adjusted analyses, findings consistent with the limitations of using a cross-sectional design with a predominantly normal-weight cohort. The high prevalence of salt addition in the sample, the large proportion of students unable to quantify their salt intake, and the detectable disease signal in such a young population highlight a dietary pattern worthy of further investigation in the Saudi university health context. Prospective studies featuring objective dietary and cardiometabolic biomarkers are essential to confirm the associations reported in this study and to inform the design and implementation of culturally appropriate, evidence-based NCD prevention programs targeting Saudi university students.

## Data Availability

The raw data supporting the conclusions of this article will be made available by the author, without undue reservation.
